# The effect of combined ergonomic training and exercises on musculoskeletal pain and ergonomic risks in supermarket cashiers: a randomized controlled trial

**DOI:** 10.1007/s00420-025-02132-z

**Published:** 2025-03-08

**Authors:** Devran Yaşar, Elif Esma Safran, Ömer Şevgin

**Affiliations:** 1 Institute of Health Sciences, Department of Physiotherapy and Rehabilitation, Üsküdar University, Istanbul, Turkey; 2 Faculty of Health Sciences, Department of Physiotherapy and Rehabilitation, Acibadem Mehmet Ali Aydinlar University, Istanbul, Turkey; 3 Faculty of Health Sciences, Department of Physiotherapy and Rehabilitation, Üsküdar University, Istanbul, Turkey

**Keywords:** Ergonomics, Exercise therapy, Musculoskeletal diseases, Occupational health, Retail workers

## Abstract

**Purpose:**

This study aimed to evaluate the combined effects of a 12-week ergonomic training and exercise program on musculoskeletal pain and ergonomic risks among supermarket cashiers.

**Methods:**

This study cohort comprised 77 cashiers, aged between 18 and 45, who were experiencing musculoskeletal pain. Of these, 60 participants completed the study after random assignment to either the intervention group, which received ergonomic training and exercise, or the control group, which received only ergonomic training. The study lasted 12 weeks, with assessments conducted at baseline (week 0) and 12 weeks post-intervention. The outcomes included the Visual Analogue Scale, the Extended Nordic Musculoskeletal Questionnaire (NMQ-E), and the Rapid Upper Limb Assessment. The clinical trial registration number is NCT06407440.

**Results:**

Musculoskeletal pain in the upper back, lower back, and hips/thighs decreased significantly after the intervention, while there were no significant changes in the control group, except for a reduction in upper back pain. A significant improvement in activity limitations was observed in the intervention group, particularly in the upper back and lower back. Consultations with health professionals for upper and lower back pain (NMQ-E) decreased significantly in the intervention group. Symptoms in the past 7 days showed a significant decrease in the intervention group, especially for the upper back, lower back, and hips/thighs, with no significant change in the control group.

**Conclusion:**

The integrated approach of ergonomic training and exercise programs has the potential to alleviate musculoskeletal discomfort among supermarket cashiers. These interventions may prove an effective strategy for enhancing the well-being of workers in physically demanding retail environments.

**Supplementary Information:**

The online version contains supplementary material available at 10.1007/s00420-025-02132-z.

## Introduction

Supermarket cashiers work in a demanding environment characterized by repetitive tasks such as scanning items and interacting with customers. Although these activities are classified as light manual labor, they are associated with significant physical and mental strain. The inherent stressors of the job, including time pressure and the need for high accuracy, contribute to psychological stress (Deng et al. 2020; Roxo et al. 2021). Cashiers are particularly vulnerable to work-related musculoskeletal disorders (WMSDs) (Algarni and Alkhaldi 2021; Choi et al. 2017), especially in the upper limbs, neck, and lower back (Silva et al. 2024), due to repetitive movements, static postures, and the physical demands of their tasks (Algarni and Alkhaldi 2021; Deng et al. 2020).

Research indicates a high prevalence of musculoskeletal disorder (MSD) symptoms among cashiers, with many experiencing discomfort in multiple body regions. Factors such as prolonged static postures and fast-paced work exacerbate this risk, particularly concerning shoulder, neck, and lower back pain (Algarni and Alkhaldi 2021; Anton and Weeks 2016; Deng et al. 2020; Silva et al. 2024). With lower back pain (LBP) being a leading cause of disability and sick leave, cashiers’ ergonomic conditions must be assessed and improved to alleviate these problems and improve their overall health and job satisfaction (Algarni and Alkhaldi 2021; Govindu and Babski-Reeves 2014).

Despite the known risks, there is limited evidence of effective interventions to reduce musculoskeletal pain in cashiers. Ergonomic training aimed at optimizing body mechanics and workstation design has been shown to be effective in reducing the risk of WMSDs in various occupations (Denadai et al. 2021; Lee et al. 2021; Sohrabi and Babamiri 2022), but there is a notable lack of research specifically focused on cashiers in the retail sector. In addition, exercise programs targeting muscle strength and flexibility are recommended as preventive measures for individuals in physically demanding jobs (Bullo et al. 2022; Tersa-Miralles et al. 2022), but their effectiveness in the retail environment, particularly for cashiers, remains underexplored.

Given the high prevalence of MSDs among supermarket cashiers and the limited research on ergonomic and exercise interventions in this specific group, randomized controlled trials (RCTs) are needed to assess the potential benefits of such interventions. This research investigates the impact of a 12-week intervention combining ergonomic training and exercises on reducing musculoskeletal pain and ergonomic risks in supermarket cashiers. Additionally, it sought to compare outcomes between the intervention and control groups to highlight the added value of this integrated approach.

## Methods

### Study design and participants

This study was a prospective, randomized controlled trial (RCT) conducted in a supermarket setting with the approval of the Medical Research Ethics Committee. Participants were verbally informed about the study, and written informed consent was obtained.

In this study, G*Power 3.1.7. software was used to determine the sample size. Based on the effect size (0.48) reported in the Lee et al. study (Lee et al. 2021), it was calculated that a minimum of 30 participants per group would be required with a 95% confidence interval and 5% margin of error. Accordingly, 60 participants were targeted to achieve 80% performance, allowing for the possibility of data loss.

Cashiers aged 18–45, who reported musculoskeletal pain in the last three months and worked more than 40 h per week, were recruited. Exclusion criteria included pregnancy, recent surgery (within the last six months), chronic illnesses affecting musculoskeletal health, and a body mass index (BMI) over 30 kg/m².

Musculoskeletal pain was assessed through validated self-report tools, including the Visual Analogue Scale (VAS) and the Extended Nordic Musculoskeletal Questionnaire (NMQ-E). Recruitment was facilitated via internal announcements, and participation was entirely voluntary. Although there was no direct collaboration with healthcare professionals or the company’s health and safety sector, all participants were thoroughly briefed on the study’s objectives.

### Randomization and blinding

Participants were randomly assigned to either the intervention group (IG) or the control group (CG) using block randomization (Random Allocation Software). Due to the nature of the intervention, neither the participants nor the researchers could be blinded to group assignment. However, outcome assessors were blinded to group allocation to minimize bias.

**Fig. 1:**
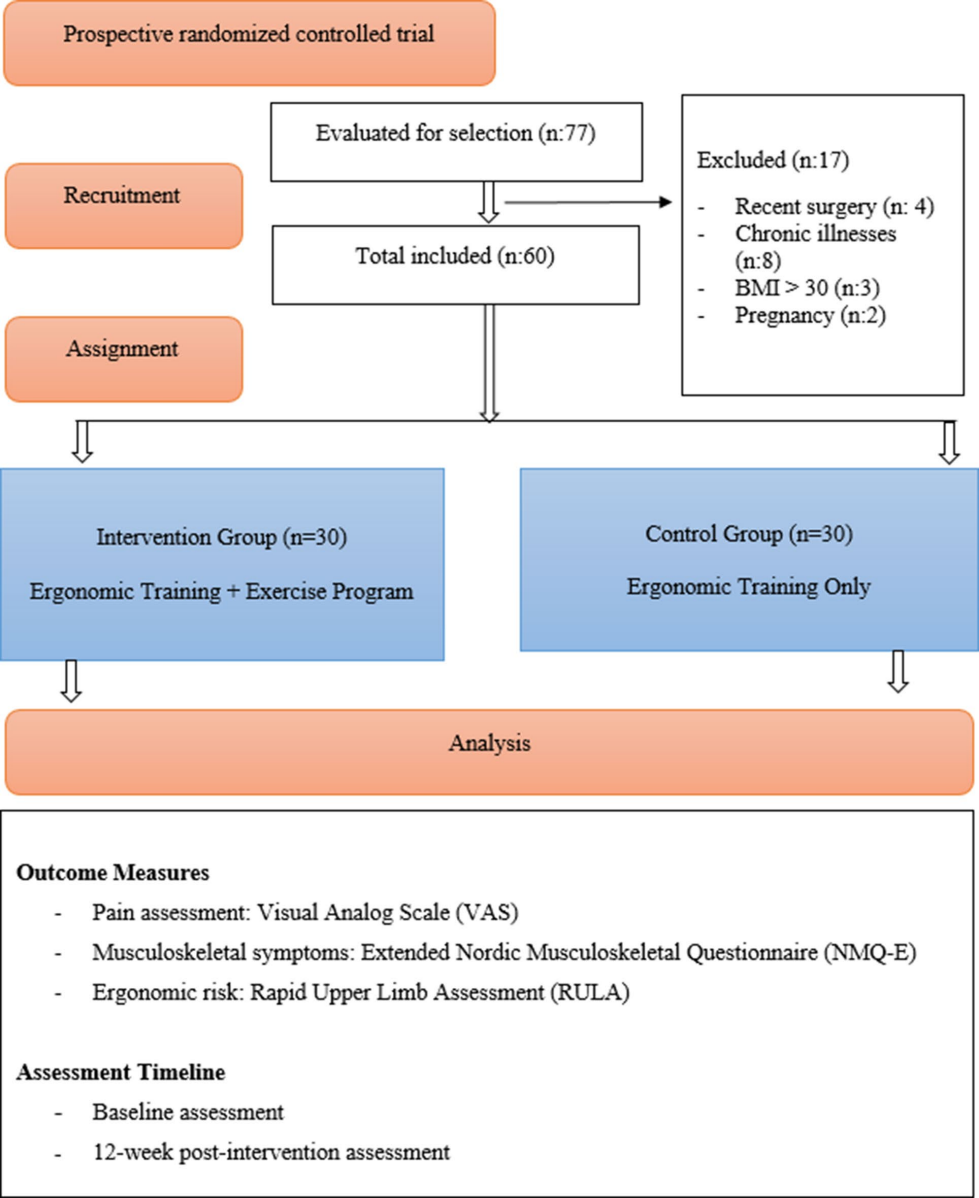
Flow chart of the study

The flowchart of the study is shown in Fig. 1. The IG received both ergonomic training and an exercise program designed to reduce musculoskeletal pain. The CG only received ergonomic training. Assessments were conducted at baseline (week 0) and 12 weeks post-intervention (end of the study) to evaluate the intervention’s immediate and cumulative effects.

### Interventions

The intervention group participated in a 12-week ergonomic training and exercise program. The program was designed to reduce musculoskeletal pain through improved workstation ergonomics and targeted physical exercise. The ergonomic training and exercise program was developed by a team of physiotherapists and supported by occupational therapists, ensuring that the program was comprehensive and tailored to the needs of the participants.

The RULA was used to identify ergonomic risks associated with cashiers’ postures and to assess the effectiveness of the intervention. While primarily focused on postural improvements, RULA also provided actionable insights for designing ergonomic interventions tailored to this occupational setting (Daneshmandi et al. 2019; Morrissey et al. 2014; Roxo et al. 2021).

#### Ergonomic training

All participants, including both the intervention and control groups, received comprehensive ergonomic training. This training included instruction on several key topics, such as what ergonomics is, the importance and goals of ergonomics, risk factors for MSDs that can occur in cashiers, ergonomic adjustments that can be made in the work environment, and methods to prevent MSDs. In addition, the training covered postural disorders, various ergonomic adjustments for these disorders, chair selection, monitor selection, and the importance of rest breaks.

Participants were instructed on correct body mechanics when sitting, standing, and reaching, and the correct positioning of the checkout workstation. Personalized advice was given on chair and footrest adjustments, tailored to each participant’s needs. The training included two interactive sessions that focused on optimizing workstation setup and improving body mechanics to minimize strain. Participants were also taught proper lifting techniques and strategies to reduce repetitive strain injuries. Educational materials, such as brochures with detailed ergonomic guidelines and recommended postures, were distributed to reinforce learning.

#### Exercise Program

The exercise training program lasted 12 weeks, with sessions conducted three times per week, each lasting around 45 min. Before starting, participants were briefed on the exercises. The exercise protocol was divided into three phases: warm-up, main exercises, and cool-down. The main exercises consisted of stretching, postural exercise, mobility, and strengthening exercises. Dynamic and static stretches, along with nerve stretches, were included in the routine. Postural strengthening and functional exercises targeting the upper extremities were also provided. Depending on the participants’ exercise capacity, the movements were modified as needed. The exercise program further included median, ulnar, and radial nerve stretches, neck and thoracic mobility exercises, and functional strength exercises for the shoulders, arms, hands, and wrists. Progress was monitored regularly through online meetings to ensure proper adherence and technique (see Appendix 1).

### Outcome measures

The socio-demographic information form is designed to collect essential information from study participants. Participants will provide their name, age, gender, telephone number, and email address for communication purposes. Employment details, including occupation, workplace, position, total years of experience, and average weekly hours worked, will be collected to assess the influence of the work environment on musculoskeletal pain. Participants will also indicate whether they have any chronic health conditions and whether they have experienced musculoskeletal pain in the past three months. In addition, questions about regular physical activity will be included to assess how lifestyle factors may affect pain levels.

#### Pain assessment

The VAS was selected for its simplicity and reliability in quantifying pain intensity, as demonstrated in both clinical and occupational health settings (Thong et al. 2018). Participants were instructed to identify the location of their pain on a body diagram. The severity of neck and back pain was measured using the VAS, where 0 represented “no pain” and 10 indicated “the worst pain imaginable.” The distance from the starting line to the point marked by the participant was measured in centimeters and recorded for analysis (El-Tallawy et al. 2021).

#### Extended Version of the Nordic Musculoskeletal Questionnaire (NMQ-E)

The NMQ is designed to assess work-related musculoskeletal symptoms and standardize the measurement of reported problems for better comparison between studies. It has a body diagram divided into nine anatomical regions: neck, shoulders, elbows, wrists/hands, upper back, lower back, hips/thighs, knees, and ankles/feet. Participants indicate the presence of pain or discomfort in the past 12 months, any problems with daily activities, visits to the doctor for symptoms, and complaints in the past seven days. Scoring is qualitative and focuses on the number of affected regions, with a higher score indicating a greater prevalence of musculoskeletal problems, thus highlighting the impact of work-related factors on health (Alaca et al. 2019; Dawson et al. 2009; Kakaraparthi et al. 2023; Le 2023).

#### Rapid upper limb assessment (RULA)

The RULA was used to conduct a rapid ergonomic assessment of cashiers based on their job tasks. RULA’s wide application in occupational health studies supports its use in identifying ergonomic risk factors (Kakaraparthi et al. 2023). RULA is specifically designed to analyze neck and upper limb strain and to assess posture, strength, and movements associated with sedentary tasks such as computer work. The ergonomic assessment uses a scoring system ranging from 1 to 7, with higher scores indicating a higher level of apparent risk. However, it is important to note that a low RULA score does not necessarily mean that the workplace is free of ergonomic hazards, and, conversely, a high score does not guarantee the severity of the ergonomic problem (Kakaraparthi et al. 2023; McAtamney and Nigel Corlett 1993; Silva et al. 2024).

#### The Pittsburgh sleep quality index (PSQI)

The PSQI was included to assess sleep quality and its relationship with musculoskeletal pain. This validated tool used to measure sleep quality and disturbances over 1 month. It assesses several dimensions of sleep, including subjective sleep quality, sleep latency, sleep duration, habitual sleep efficiency, sleep disturbances, use of sleeping medication, and daytime dysfunction. The PSQI produces a global score ranging from 0 to 21, with higher scores indicating poorer sleep quality. A global score greater than 5 indicates clinically significant sleep disturbance (Agargun 1996; Buysse et al. 1989). The PSQI was selected for this study due to its robust psychometric properties and high reliability across diverse populations. Recent studies have shown a strong correlation between poor sleep quality and increased musculoskeletal pain severity, making the PSQI a necessary component of this study (Gao et al. 2024; Zitser et al. 2022). Understanding these interactions is essential to address holistic health outcomes for cashiers.

### Data analysis

Descriptive statistics (means and standard deviations) were used to summarize the demographic, clinical, and work-related characteristics of the participants. Categorical variables were presented as frequencies and percentages. Normality of the data was assessed using the skewness and kurtosis coefficients, where values between − 2 and + 2 were considered indicative of normal distribution according to George and Mallery (George and Mallery 2019).

For comparisons between groups, the Mann-Whitney U test was used for nonparametric data, while independent t-tests were used for continuous variables with a normal distribution. The chi-squared test was used for proportional data comparisons. The Wilcoxon signed-rank test was used to assess within-group changes over time (before and after the intervention).

Skewness and kurtosis analyses revealed that VAS and RULA data followed a normal distribution; hence, parametric tests, such as the independent t-test, were used for between-group comparisons. Conversely, the NMQ-E and PSQI data did not meet the assumption of normality, and nonparametric tests, such as the McNemar test, were employed for within-group analyses. Differences in the prevalence of musculoskeletal pain in different body regions before and after the intervention were also analyzed using the McNemar test.

Spearman’s rank correlation was used to explore relationships between variables. All statistical analyses were performed using IBM SPSS version 21.0 (IBM Corp., Armonk, NY, USA), and statistical significance was set at *p* < 0.05.

## Results

Changes in musculoskeletal pain and ergonomic risks were observed between baseline and 12 weeks post-intervention. Intermediate evaluations were not included to streamline data collection and analysis.

### Demographic characteristics

Table 1 compares the demographic, clinical, and work-related characteristics of the intervention (IG) and control (CG) groups. The mean age was 34.44 years for IG and 33.64 years for CG, with no significant difference (*p* = 0.582). Both groups had a balanced distribution of male and female participants (*p* = 0.297). There was no significant difference in BMI between the groups (*p* = 0.318). IG participants had 2–12 years of experience and CG participants had 2–23 years, with no significant difference (*p* = 0.428). Both groups worked 48–60 h per week, with no difference (*p* = 0.958). Conditions such as hypertension, diabetes, and lumbar disc herniation were present in IG, while CG had hypertension, migraine, and osteoarthritis, with no significant difference between groups (*p* = 0.141). Sleep quality was similar between groups (*p* = 0.756).

**Table 1:** Demographic, clinical, and work characteristics of the intervention and control groups

		IG (*n* = 30)	CG (*n* = 30)	*p* value
Age (years)^b^		34.44 ± 5.545	33.64 ± 5.640	0.582
Gender (n,%)^a^	Woman	11 (36.7%)	15 (50.0%)	0.297
	Male	19 (63.3%)	15 (50.0%)	
BMI (kg/m^2^)^b^		26.54 ± 3.310	26.47 ± 2.406	0.318
Work years^a^		2–12	2–23	0.428
Weekly working hours^b^		48–60	48–60	0.958
Chronic disorder^a^	Yes	8 (26.7%)	10 (33.3%)	0.141
	No	22 (73.3%)	20 (66.7%)	
Sleep quality (PSQI)^b^		1.10 ± 0.88	1.03 ± 0.76	0.756

Values are presented as mean ± standard deviation (SD) for continuous variables and n (%) for categorical variables

Abbreviations: IG: intervention group, CG: control group; BMI: body mass index; PSQI: Pittsburgh Sleep Quality Index

Statistical significance was defined as p < 0.05., a: Mann-Whitney U Test, b: Independent Groups T-Test

## Comparison of pain severity (VAS), ergonomic risk (RULA) and sleep quality (PSQI) scores

Table 2 shows the comparison of pre-and post-intervention VAS, RULA, and PSQI scores for both within-group and between-group assessments.

**Table 2:** Comparison of pre- and post-intervention VAS, RULA, and PSQI scores for within-group and between-group assessments

Variable	Group	*n*	Mean Pre	Mean Post	t (Intra)	*p* (Intra)	Mean Diff (Inter)	t (Inter)	*p* (Inter)	Effect Size (Inter)
VAS	IG	30	4.87 ± 2.61	4.00 ± 2.29	0.449	0.655	0.00	0.00	1.00	0.00
	CG	30	4.00 ± 1.62	4.60 ± 1.94						
RULA	IG	30	6.03 ± 1.22	4.37 ± 1.27	0.332	0.741	-0.40	-1.30	0.199	0.336
	CG	30	4.77 ± 1.10	5.93 ± 1.11						
PSQI	IG	30	1.10 ± 0.88	1.27 ± 1.11	-1.720	0.096	0.03	0.00	0.756	0.314
	CG	30	1.03 ± 0.76	1.10 ± 0.76	-0.812	0.423	0.10	0.00		

Values are presented as mean ± standard deviation (SD) for pre- and post-intervention measures

Abbreviations: IG: intervention group, CG: control group; VAS: Visual Analog Scale; RULA: Rapid Upper Limb Assessment; PSQI: Pittsburgh Sleep Quality Index

Intra-group comparisons (t and p values) assess changes within each group (IG: intervention group, CG: control group) over time

The between-group comparisons (mean difference, t, p values and effect size) reflect the differences between the two groups on post-intervention measures

Statistical significance was defined as *p* < 0.05

### VAS scores

In the intra-group comparison, the intervention group (IG) showed a decrease in pain severity from 4.87 ± 2.61 (pre-intervention) to 4.00 ± 2.29 (post-intervention), although this change was not statistically significant (*p* = 0.655). Similarly, in the control group (CG), pain scores increased slightly from 4.00 ± 1.62 to 4.60 ± 1.94, with no significant change (*p* = 1.00).

When comparing between groups, the mean difference in post-intervention VAS scores between IG and CG was 0.00, indicating no significant difference between groups (*p* = 1.00, effect size = 0.00).

### RULA scores

Within group comparison, the IG showed an improvement in ergonomic posture, with scores decreasing from 6.03 ± 1.22 to 4.37 ± 1.27. However, this improvement was not statistically significant (*p* = 0.741). In contrast, the CG showed an increase in ergonomic risk, with scores increasing from 4.77 ± 1.10 to 5.93 ± 1.11.

When compared between groups, the mean difference in post-intervention RULA scores between IG and CG was − 0.40, with no statistically significant difference between groups (*p* = 0.199, effect size = 0.336).

### PSQI scores

In the intra-group comparison, the IG’s PSQI scores increased slightly from 1.10 ± 0.88 to 1.27 ± 1.11, but this change was not statistically significant (*p* = 0.096). Similarly, the CG’s scores showed a minimal increase from 1.03 ± 0.76 to 1.10 ± 0.76, with no significant change (*p* = 0.423).

When comparing between groups, the mean difference in post-intervention PSQI scores between IG and CG was 0.03, indicating no significant difference between groups (*p* = 0.756, effect size = 0.314).

### Pain in different parts of the musculoskeletal system

The comparison of musculoskeletal pain in different body regions before and after the intervention, based on NMQ-E, is shown in Table 3. Significant improvements were observed in the IG after ergonomic training and exercise, especially in the upper back, lower back, and hips/thighs.

**Table 3:** Comparison of musculoskeletal pain in different body regions pr- and pos- intervention (NMQ-E results)

		IG			CG		
		Pre *n* (%)	Post *n* (%)	*p* value	Pre *n* (%)	Post *n* (%)	*p* value
Ache, pain, discomfort tingling, or numbness in the past 12 months	Neck	20 (66.6)	19 (63.27)	1	14 (46.62)	12 (39.96)	0.852
	Shoulders	2 (6.66)	1 (3.33)	0.250	3 (9.99)	3 (9.99)	1
	Upper back	16 (53.28)	9 (29.97)	0.035*	12 (39.96)	4 (13.32)	0.035*
	Elbows	0	0	1	0	0	1
	Wrists/Hands	2 (6.66)	1 (3.33)	0.250	0	0	1
	Lower back	17 (56.61)	10 (33.3)	0.036*	8 (26.64)	6 (19.98)	0.760
	Hips/Thighs	10 (33.3)	4 (13.32)	0.043*	4 (13.32)	3 (9.99)	1
	Knees	2 (6.66)	3 (9.99)	1	3 (9.99)	3 (9.99)	1
	Ankles/Feet	2 (6.66)	2 (6.66)	1	8 (26.64)	7 (23.31)	1
Prevention of carrying out normal activities in the past 12 months	Neck	17 (56.61)	13 (43.29)	0.0625	13 (43.29)	14 (46.62)	1
	Shoulders	3 (9.99)	2 (6.66)	1	3 (9.99)	4 (13.32)	1
	Upper back	14 (46.62)	7 (23.31)	0.0230*	11 (36.63)	4 (13.32)	0.033*
	Elbows	1 (3.33)	1 (3.33)	1	0	0	1
	Wrists/Hands	1 (3.33)	1 (3.33)	1	1 (3.33)	1 (3.33)	1
	Lower back	10 (33.3)	7 (23.31)	0.032*	7 (23.31)	4 (13.32)	0.075
	Hips/Thighs	3 (9.99)	2 (6.66)	1	2 (6.66)	3 (9.99)	1
	Knees	0	0	1	2 (6.66)	2 (6.66)	1
	Ankles/Feet	1 (3.33)	1 (3.33)	1	4 (13.32)	4 (13.32)	1
Consult a health professional in the past 12 months	Neck	16 (53.28)	13 (43.29)	0.780	13 (43.29)	9 (29.97)	0.625
	Shoulders	3 (9.99)	3 (9.99)	1	3 (9.99)	4 (13.32)	1
	Upper back	13 (43.29)	9 (29.97)	0.045*	11 (36.63)	9 (29.97)	0.920
	Elbows	0	0	1	0	0	1
	Wrists/Hands	2 (6.66)	1 (3.33)	1	1 (3.33)	2 (6.66)	1
	Lower back	9 (29.97)	4 (13.32)	0,024*	7 (23.31)	6 (19.98)	1
	Hips/Thighs	3 (9.99)	2 (6.66)	1	1 (3.33)	3 (9.99)	0.750
	Knees	1 (3.33)	1 (3.33)	1	3 (9.99)	2 (6.66)	1
	Ankles/Feet	1 (3.33)	1 (3.33)	1	7 (23.31)	8 (26.64)	1
Symptom or trouble in the past 7 days	Neck	22 (73.26)	19 (63.27)	0.820	12 (39.96)	14 (46.62)	0.760
	Shoulders	4 (13.32)	4 (13.32)	1	2 (6.66)	2 (6.66)	1
	Upper back	7 (23.31)	2 (6.66)	0.025*	7 (23.31)	3 (9.99)	0.045*
	Elbows	1 (3.33)	0	0.350	0	0	1
	Wrists/Hands	2 (6.66)	3 (9.99)	1	0	0	1
	Lower back	14 (46.62)	5 (16.65)	0.018*	5 (16.65)	4 (13.32)	1
	Hips/Thighs	7 (23.31)	2 (6.66)	0.025*	2 (6.66)	2 (6.66)	1
	Knees	2 (6.66)	2 (6.66)	1	2 (6.66)	2 (6.66)	1
	Ankles/Feet	0	1 (3.33)	1	6 (19.98)	6 (19.98)	1

Values are presented as n (%)

Abbreviations: IG: intervention group, CG: control group; NMQ-E: Extended Version of the Nordic Musculoskeletal Questionnaire

Intra-group comparisons (p values) assess changes within each group (IG: intervention group, CG: control group) over time using the McNemar test

Statistical significance was defined as *p* < 0.05

In the neck region, the prevalence of musculoskeletal pain decreased slightly in the IG from 66.6% before the intervention to 63.27% after the intervention, although this change was not statistically significant (*p* = 1.000). The CG showed a smaller reduction from 46.62 to 39.96%, which was also not significant (*p* = 0.852). For the shoulders, no significant changes were observed in either group.

In contrast, the upper back region showed a significant reduction in pain prevalence in the IG, from 53.28 to 29.97% post-intervention, which was statistically significant (*p* = 0.035). A similar pattern was observed in the CG, where pain prevalence decreased from 39.96 to 13.32% (*p* = 0.035). The IG also showed significant decreases in the prevalence of low back pain (from 56.61 to 33.3%, *p* = 0.036) and hip/thigh pain (from 33.3 to 13.32%, *p* = 0.043). No statistically significant changes were found in other body regions, including elbows, wrists/hands, knees, and ankles/feet.

The impact of musculoskeletal pain on daily activities was also assessed. The IG showed significant improvements in the upper back (*p* = 0.023) and lower back (*p* = 0.032), with reduced reports of activity limitation after the intervention. The CG showed similar trends for the upper back (*p* = 0.033), although no other significant differences in activity limitations were observed.

In terms of consultations with health professionals, significant reductions were noted in the IG for the upper back (*p* = 0.045) and lower back (*p* = 0.024) post-intervention. The CG did not show any significant changes in consultations with health professionals for any of the body regions.

Finally, in terms of symptoms experienced in the past 7 days, the IG showed a statistically significant decrease in the upper back (*p* = 0.025), lower back (*p* = 0.018), and hips/thighs (*p* = 0.025). The CG showed a significant reduction only in the upper back (*p* = 0.045).

## Discussion

This study aimed to evaluate the effectiveness of a 12-week ergonomic training and exercise program in reducing musculoskeletal pain in supermarket cashiers. The IG, which received both ergonomic training and exercise, demonstrated a significant reduction in musculoskeletal pain in the upper, lower back, and hips/thighs compared with the CG, highlighting the targeted benefits of the intervention. However, sleep quality, as measured by the PSQI, showed minimal changes post-intervention in both the intervention and control groups. This suggests that while the physical interventions effectively reduced musculoskeletal pain, their impact on sleep quality was limited.

While the combined ergonomic training and exercise intervention was hypothesized to yield superior results compared to the control group, the study aimed to quantify the magnitude and scope of these improvements. This approach provides evidence-based insights into the practical benefits of such interventions, helping to establish their applicability in occupational settings.

The findings align with previous literature emphasizing the effectiveness of ergonomic interventions in mitigating WMSDs across various professions. Dandale et al. (Dandale et al. 2023) and Kakaraparthi et al. (Kakaraparthi et al. 2023) highlighted similar benefits in reducing chronic pain and improving posture. In occupations involving repetitive tasks and static postures, such as office workers and factory employees, combined ergonomic adjustments and exercises led to significant pain reduction (Pehlevan and Şevgin 2024; Sundstrup et al. 2020). These consistent results affirm the broader applicability of integrated ergonomic strategies.

The use of RULA and VAS tools provided valuable insights into ergonomic risk and pain severity. However, the lack of significant changes in RULA scores suggests limitations in detecting subtle ergonomic improvements in dynamic retail settings (Martinez-Silveira and Fernandes 2024). Similarly, while regional pain reductions were observed, overall VAS scores showed no significant differences between IG and CG, indicating the need for broader or longer-term interventions.

The findings of the scoping review by Grana et al. (Grana et al. 2020) emphasize the value of combining education and physical interventions, such as seminars and exercises, to manage musculoskeletal pain, particularly in the back, neck and upper limbs. This supports our study’s conclusion that combining ergonomic training with exercise is particularly effective in addressing pain in these region. A notable limitation across studies, including those reviewed by Grana et al. (Grana et al. 2020), and Martinez-Silveira et al. (Martinez-Silveira and Fernandes 2024), is the lack of long-term follow-up, which underscores the need for extended evaluation to assess the sustainability of benefits.

The NMQ-E has proven to be a reliable tool for assessing musculoskeletal symptoms across various occupational groups, including office and retail workers (Algarni and Alkhaldi 2021; Dawson et al. 2009). Its application in this study validated its utility in retail settings, providing robust insights into musculoskeletal outcomes in high-risk environments. This aligns with previous studies demonstrating reductions in reported symptoms following ergonomic interventions in office settings (Algarni and Alkhaldi 2021; Hailu Tesfaye et al. 2023).

Musculoskeletal pain is a common problem in occupations that involve frequent use of the upper extremities, such as dentistry (Eminoğlu et al. 2024), cosmetology (Acar and Acımıs 2023), and cashiering (Algarni and Alkhaldi 2021; Algarni et al. 2020; Deng et al. 2020; Roxo et al. 2021; Silva et al. 2024; Tesfaye et al. 2023), due to repetitive movements and prolonged postures. The reduction in pain observed in this study mirrors findings in other high-risk groups. For example, Alseminy et al. (Alseminy et al. 2022) reported that increased physical activity was associated with better musculoskeletal outcomes among young adults. This highlights the value of incorporating exercise and ergonomic education as part of workplace interventions to reduce musculoskeletal pain and improve worker health.

The relationship between sleep quality and musculoskeletal health is well-documented. Poor sleep quality has been shown to exacerbate pain perception and delay recovery from musculoskeletal disorders (Gao et al. 2024; Zitser et al. 2022). In this study, the interventions led to notable pain relief in musculoskeletal regions. However, no significant changes were noted in PSQI scores, suggesting that while these interventions effectively address physical pain, their impact on sleep quality may be limited. Given the bidirectional relationship between pain and sleep, future studies could explore targeted approaches to address sleep disturbances alongside ergonomic and exercise programs.

The integration of exercise programs into ergonomic interventions provides not only physical relief but also long-term benefits, as demonstrated in studies highlighting reductions in workplace absenteeism, improved muscular endurance, and sustained ergonomic improvements (Kakaraparthi et al. 2023; Pehlevan and Şevgin 2024; Sundstrup et al. 2020). Additionally, Pereira et al. (Pereira et al. 2019) found that combining workplace ergonomics with targeted exercise programs reduced neck pain and sickness absenteeism in office workers, highlighting the long-term applicability of such interventions in reducing work-related strain. The consistency of these findings across occupations reinforces the effectiveness of a combined intervention approach, particularly in occupations that involve repetitive use of the upper extremities.

### Strengths and limitations

This study’s primary strength lies in its focus on a high-risk occupational group—supermarket cashiers—and its use of a randomized controlled design to evaluate tailored interventions. By combining ergonomic training with an exercise program, the study addressed multiple risk factors for WMSDs, such as poor posture, repetitive strain, and lack of physical conditioning. While the combined ergonomic training and exercise intervention was hypothesized to yield superior results compared to the control group, the study’s goal was to quantify the magnitude and scope of these improvements. This approach provides evidence-based insights into the practical benefits of such interventions, helping to establish their applicability in occupational health settings. This comprehensive approach is particularly relevant given cashiers’ exposure to prolonged static postures and repetitive arm movements.

Conducting the study in a uniform workplace environment allowed for controlled assessment of intervention effects. However, reliance on self-reported pain measures and exclusion of participants with high BMI may limit generalizability. Incorporating objective health measures and broader participant demographics in future studies could provide more comprehensive insights.

However, the study has limitations. The exclusion of participants with a BMI over 30 kg/m², while necessary to control for obesity’s confounding effects on musculoskeletal pain, limits the applicability of the findings to populations with higher BMI values. Additionally, the relatively small sample size may restrict the generalizability of the results. The reliance on self-reported pain assessments, such as the Visual Analogue Scale (VAS), introduces potential bias, and the lack of long-term follow-up (Grana et al. 2020) leaves the sustainability of the observed benefits uncertain. Furthermore, the lack of qualitative analysis limited insights into participants’ subjective experiences with the intervention, and the use of basic statistical methods may have constrained the depth of time-dependent analysis.

Future research should address these limitations by incorporating larger, more diverse samples, objective measures of musculoskeletal health, extended follow-up periods, qualitative approaches to understand participant perspectives, and advanced statistical techniques, such as mixed-effects modeling, to better evaluate intervention outcomes over time.

## Conclusion

This study aimed to evaluate the effectiveness of a 12-week ergonomic training and exercise program in reducing musculoskeletal pain among supermarket cashiers. The intervention group showed significant reductions in pain intensity, particularly in the upper back, lower back, and hips/thighs, as well as improved activity limitations compared to the control group. Additionally, consultations with health professionals and reports of recent symptoms decreased significantly in the intervention group, highlighting the potential of these interventions to alleviate workplace-related musculoskeletal discomfort. Furthermore, the comparison between the intervention and control groups underscores the added value of combining ergonomic training with exercise, providing evidence-based support for integrated interventions in occupational settings.

However, the study’s relatively short duration and lack of long-term follow-up limit the generalizability of its findings. Future studies should focus on assessing the sustainability of these benefits over longer periods and exploring the applicability of similar interventions in other occupational settings.

## Electronic supplementary material

Below is the link to the electronic supplementary material.

Supplementary Material 1

## Data Availability

Data available on request due to privacy/ethical restrictions.

## References

[CR1] Acar GA, Acımıs NM (2023) Neck and upper extremity musculoskeletal problems in cosmetologists caused by work-related ergonomic risk factors in Denizli, Turkey. Work 75(3):953–964 36591680 10.3233/WOR-220056

[CR2] Agargun MY (1996) Pittsburgh Uyku kalitesi Indeksinin gecerligi ve guvenirligi. Turk Psikiyatri Dergisi 7:107–115

[CR3] Alaca N, Safran EE, Karamanlargil AI, Timucin E (2019) Translation and cross-cultural adaptation of the extended version of the nordic musculoskeletal questionnaire into Turkish. J Musculoskel Neuronal Interact 19(4):472–481 PMC6944807 31789298

[CR4] Algarni FS, Alkhaldi HA (2021) Literature review of musculoskeletal disorders and their risk factors among supermarket cashiers. Rehabilitation Sci 6(2):25–40. 10.11648/j.rs.20210602.12

[CR5] Algarni FS, Alkhaldi HA, Zafar H, Kachanathu SJ, Al-Shenqiti AM, Altowaijri AM (2020) Self-reported musculoskeletal disorders and quality of life in supermarket cashiers. Int J Environ Res Public Health 17(24):9256 33322079 10.3390/ijerph17249256 PMC7763189

[CR6] Alseminy MAMM, Chandrasekaran B (2022) Bairapareddy KC Association of physical activity and quality of life with work-related musculoskeletal disorders in the UAE young adults. In: Healthcare, vol 10. MDPI, p 625 10.3390/healthcare10040625 PMC9028778 35455803

[CR7] Anton D, Weeks DL (2016) Prevalence of work-related musculoskeletal symptoms among grocery workers. Int J Ind Ergon 54:139–145. 10.1016/j.ergon.2016.05.006

[CR8] Bullo V et al (2022) Resistance training improves physical fitness and reduces pain perception in workers with upper limb Work-Related musculoskeletal disorders: A pilot study. Hygiene 2(3):136–145. 10.3390/hygiene2030012

[CR9] Buysse DJ, Reynolds CF, Monk TH, Berman SR, Kupfer DJ (1989) The Pittsburgh sleep quality Index - a new instrument for psychiatric practice and research. Psychiat Res 28(2):193–213 doi:Doi 10.1016/0165–1781(89)90047-4 10.1016/0165-1781(89)90047-4 2748771

[CR10] Choi HW, Kim YK, Kang DM, Kim JE, Jang BY (2017) Characteristics of occupational musculoskeletal disorders of five sectors in service industry between 2004 and 2013. Ann Occup Environ Med 29:41. 10.1186/s40557-017-0198-4 28936358 10.1186/s40557-017-0198-4 PMC5604364

[CR11] Dandale C, Telang PA, Kasatwar P (2023) The effectiveness of ergonomic training and therapeutic exercise in chronic neck pain in accountants in the healthcare system: A review. Cureus 15(3):e35762. 10.7759/cureus.35762 37025734 10.7759/cureus.35762 PMC10072180

[CR12] Daneshmandi H, Kee D, Kamalinia M, Oliaei M, Mohammadi H (2019) An ergonomic intervention to relieve musculoskeletal symptoms of assembly line workers at an electronic parts manufacturer in Iran. Work 61(4):515–521 10.3233/WOR-182822 30475781

[CR13] Dawson AP, Steele EJ, Hodges PW, Stewart S (2009) Development and test-retest reliability of an extended version of the nordic musculoskeletal questionnaire (NMQ-E): a screening instrument for musculoskeletal pain. J Pain 10(5):517–526. 10.1016/j.jpain.2008.11.008 19345154 10.1016/j.jpain.2008.11.008

[CR14] Denadai MS, Alouche SR, Valentim DP, Padula RS (2021) An ergonomics educational training program to prevent work-related musculoskeletal disorders to novice and experienced workers in the poultry processing industry: A quasi-experimental study. Appl Ergon 90:103234. 10.1016/j.apergo.2020.103234 32932013 10.1016/j.apergo.2020.103234

[CR15] Deng M, Wu F, Luan F (2020) Musculoskeletal disorders, psychological distress, and work error of supermarket cashiers. Hum Factors Ergon Manuf Serv Ind 30(1):22–28

[CR16] El-Tallawy SN, Nalamasu R, Salem GI, LeQuang JAK, Pergolizzi JV, Christo PJ (2021) Management of musculoskeletal pain: an update with emphasis on chronic musculoskeletal pain. Pain Ther 10(1):181–209. 10.1007/s40122-021-00235-2 33575952 10.1007/s40122-021-00235-2 PMC8119532

[CR17] Eminoğlu DÖ, Kaşali K, Şeran B, Burmaoğlu GE, Aydin T, Bircan HB (2024) An assessment of musculoskeletal disorders and physical activity levels in dentists: A cross-sectional study. Work(Preprint):1–11 10.3233/WOR-240067 39213120

[CR18] Gao X, Qiao Y, Chen Q, Wang C, Zhang P (2024) Effects of different types of exercise on sleep quality based on Pittsburgh sleep quality index in middle-aged and older adults: a network meta-analysis. J Clin Sleep Med 20(7):1193–1204. 10.5664/jcsm.11106 38450497 10.5664/jcsm.11106 PMC11217626

[CR19] George D, Mallery P (2019) IBM SPSS Statistics 26 Step by Step, 16th Edition edn. Routledge, New York

[CR20] Govindu NK, Babski-Reeves K (2014) Effects of personal, psychosocial and occupational factors on low back pain severity in workers. Int J Ind Ergon 44(2):335–341. 10.1016/j.ergon.2012.11.007

[CR21] Grana E, Velonakis E, Tziaferi S, Sourtzi P (2020) Effectiveness of intervention programs to manage musculoskeletal disorders at the workplace-Scoping review. Nursing Care & Research. /Nosileia kai Ereuna(57)

[CR22] Hailu Tesfaye A, Desye B, Engdaw GT (2023) Prevalence and risk factors of work-related musculoskeletal disorders among cashiers in small-scale businesses: a cross-sectional study in Ethiopia. BMJ Open 13(7):e070746. 10.1136/bmjopen-2022-070746 10.1136/bmjopen-2022-070746 PMC10360433 37474194

[CR23] Kakaraparthi VN et al (2023) Clinical application of rapid upper limb assessment and nordic musculoskeletal questionnaire in Work-Related musculoskeletal disorders: A bibliometric study. Int J Environ Res Public Health 20(3):1932. 10.3390/ijerph20031932 36767293 10.3390/ijerph20031932 PMC9914731

[CR24] Le TTT (2023) Test-retest reliability of the nordic musculoskeletal questionnaire (NMQ) in Vietnamese physical therapists. TẠp ChÍ Khoa học trường ĐẠi học quốc. TẾ HỒng BÀng 4:81–86. 10.59294/hiujs.4.2023.389

[CR25] Lee S, FC DEB, CSM DEC, T DEOS (2021) Effect of an ergonomic intervention involving workstation adjustments on musculoskeletal pain in office workers-a randomized controlled clinical trial. Ind Health 59(2):78–85. 10.2486/indhealth.2020-0188 33250456 10.2486/indhealth.2020-0188 PMC8010160

[CR26] Martinez-Silveira MS, Fernandes RCP (2024) Workplace interventions to prevent musculoskeletal disorders: a systematic review of randomized trials. Revista Brasileira De Saúde Ocupacional 49:e12. 10.1590/2317-6369/33622en2024v49e12

[CR27] McAtamney L, Nigel Corlett E (1993) RULA: a survey method for the investigation of work-related upper limb disorders. Appl Ergon 24(2):91–99. 10.1016/0003-6870(93)90080-s 15676903 10.1016/0003-6870(93)90080-s

[CR28] Morrissey M, Baird A, Sims R (2014) Impact of a multi-component participatory ergonomic intervention on work posture, psychosocial and physical risk factors associated with mobile tablet computer workstations: A controlled study. Int J Occup Health Public Health Nurs 1(3):43–69

[CR29] Pehlevan E, Şevgin O (2024) Effect of exercise given to factory workers with ergonomics training on pain and functionality: A randomized controlled trial. Work 78(1):195–205. 10.3233/WOR-230663 38701125 10.3233/WOR-230663

[CR30] Pereira M et al (2019) The impact of workplace ergonomics and neck-specific exercise versus ergonomics and health promotion interventions on office worker productivity: A cluster-randomized trial. Scand J Work Environ Health 45(1):42–52. 10.5271/sjweh.3760 30132008 10.5271/sjweh.3760

[CR31] Roxo LC, Ramos GC, Arruda ZM, Dias AC (2021) Work activity and musculoskeletal symptoms in female cashiers. Rev Bras Med Trab 19(3):324–331. 10.47626/1679-4435-2021-615 35774767 10.47626/1679-4435-2021-615 PMC9137857

[CR32] Silva TT, Sousa C, Colim A, Rodrigues MA (2024) Understanding Musculoskeletal Loadings among Supermarket Checkout Counter Cashiers: A Biomechanical Analysis. Safety 10(1):21 doi:ARTN 21 10.3390/safety10010021

[CR33] Sohrabi MS, Babamiri M (2022) Effectiveness of an ergonomics training program on musculoskeletal disorders, job stress, quality of work-life and productivity in office workers: a quasi-randomized control trial study. Int J Occup Saf Ergon 28(3):1664–1671. 10.1080/10803548.2021.1918930 33870873 10.1080/10803548.2021.1918930

[CR34] Sundstrup E, Seeberg KGV, Bengtsen E, Andersen LL (2020) A systematic review of workplace interventions to rehabilitate musculoskeletal disorders among employees with physical demanding work. J Occup Rehabil 30(4):588–612. 10.1007/s10926-020-09879-x 32219688 10.1007/s10926-020-09879-x PMC7716934

[CR35] Tersa-Miralles C, Bravo C, Bellon F, Pastells-Peiro R, Rubinat Arnaldo E, Rubi-Carnacea F (2022) Effectiveness of workplace exercise interventions in the treatment of musculoskeletal disorders in office workers: a systematic review. BMJ Open 12(1):e054288. 10.1136/bmjopen-2021-054288 10.1136/bmjopen-2021-054288 PMC8804637 35105632

[CR36] Tesfaye AH, Desye B, Engdaw GT (2023) Prevalence and risk factors of work-related musculoskeletal disorders among cashiers in small-scale businesses: a cross-sectional study in Ethiopia. BMJ Open 13(7):e070746 10.1136/bmjopen-2022-070746 PMC10360433 37474194

[CR37] Thong ISK, Jensen MP, Miró J, Tan G (2018) The validity of pain intensity measures: what do the NRS, VAS, VRS, and FPS-R measure? Scandinavian J Pain 18(1):99–107. 10.1515/sjpain-2018-0012 10.1515/sjpain-2018-0012 29794282

[CR38] Zitser J et al (2022) Pittsburgh sleep quality index (PSQI) responses are modulated by total sleep time and wake after sleep onset in healthy older adults. PLoS ONE 17(6):e0270095. 10.1371/journal.pone.0270095 35749529 10.1371/journal.pone.0270095 PMC9232154

