# Supplementary material for: The effect of combined ergonomic training and exercises on musculoskeletal pain and ergonomic risks in supermarket cashiers: a randomized controlled trial
Source: Int Arch Occup Environ Health. 2025 Mar 8;98(2):255–65. doi: 10.1007/s00420-025-02132-z (PMC11937115; doi:10.1007/s00420-025-02132-z)
Supplement: Supplementary file 1 — Supplementary Material 1 [file 420_2025_2132_MOESM1_ESM.docx]

**Slide Contents:**

**Title: Ergonomic Training and Exercise Program for Cashiers**

1. **Introduction to ergonomics**
   - Definition and importance of ergonomics in the workplace.
   - Aims of ergonomic interventions.
2. **Key topics covered in training**
   - Risk factors for musculoskeletal disorders (MSDs) specific to cashiers.
   - Ergonomic adjustments to workstations and the work environment.
   - Methods to prevent MSDs: correct body mechanics when sitting, standing and reaching.
3. **Interactive training elements**
   - Personalised advice for workstation adjustments (chair and footrest).
   - Correct lifting techniques to reduce repetitive strain.
   - Importance of rest breaks.
4. **Exercise program overview**
   - 12-week plan: frequency and session details.
   - Types of exercises: stretching, strengthening, mobility, and postural exercises.
   - Modifications according to exercise capacity and monitoring progress.
5. **Educational materials**
   - Brochures with detailed ergonomic guidelines.
   - Recommended postures and workstation set-up tips.

### Brochure Contents:

**Title: Ergonomic Guidelines for Cashiers**

**Section 1: Understanding Ergonomics**

- Ergonomics is the science of designing the workstation to fit the worker, rather than physically forcing the worker to fit the workstation.
- The benefits of ergonomics include reducing the risk of injury, improving comfort and increasing productivity.

**Section 2: Key Risk Factors for MSDs**

- Prolonged sitting or standing.
- Repetitive movements such as scanning objects.
- Poor posture or poorly designed workstations.

**Section 3: Workstation Adjustments**

- **Chair adjustments:** Adjust the height of your chair so that your feet are flat on the floor or on a footrest. Your thighs should be parallel to the floor.
- **Footrest use:** If your feet do not reach the floor, use a footrest to support them comfortably.
- **Checkout counter set-up:** Make sure the checkout counter is at a comfortable height for scanning without bending or twisting excessively.

**Section 4: Posture Tips**

- **Sitting posture:** Sit back in your chair with support for your lower back. Keep your shoulders relaxed and your elbows close to your body.
- **Standing posture:** Stand with your weight evenly distributed. Avoid locking your knees.
- **Reach:** Keep frequently used items within arm’s reach to avoid excessive stretching or twisting.

**Section 5: Proper Body Mechanics**

- **Lifting techniques:** Bend your knees, not your back, when lifting objects. Hold objects close to your body to reduce strain.
- **Rest breaks:** Take regular short breaks to stretch and relax your muscles.

**Section 6: Exercise Tips**

- **Stretching:** Perform dynamic and static stretches for the neck, shoulders, and back to reduce stiffness.
- **Strengthen:** Incorporate exercises that target the upper body, especially the shoulders, arms, and wrists, to support daily tasks.

**Contact us for more support:**

- If you have any questions or need personalized advice, please contact us.
